# Supplementary material for: Identifying repeat domains in large genomes
Source: Genome Biol. 2006 Jan 31;7(1):R7. doi: 10.1186/gb-2006-7-1-r7 (PMC1431705; doi:10.1186/gb-2006-7-1-r7)
Supplement: Additional File 1 — A zipped file of browsable HTML files with a complete list of the connected components in the repeat domain graph of human Repbase. [file gb-2006-7-1-r7-S1.gz › html/subgraphs/8328.pdf]

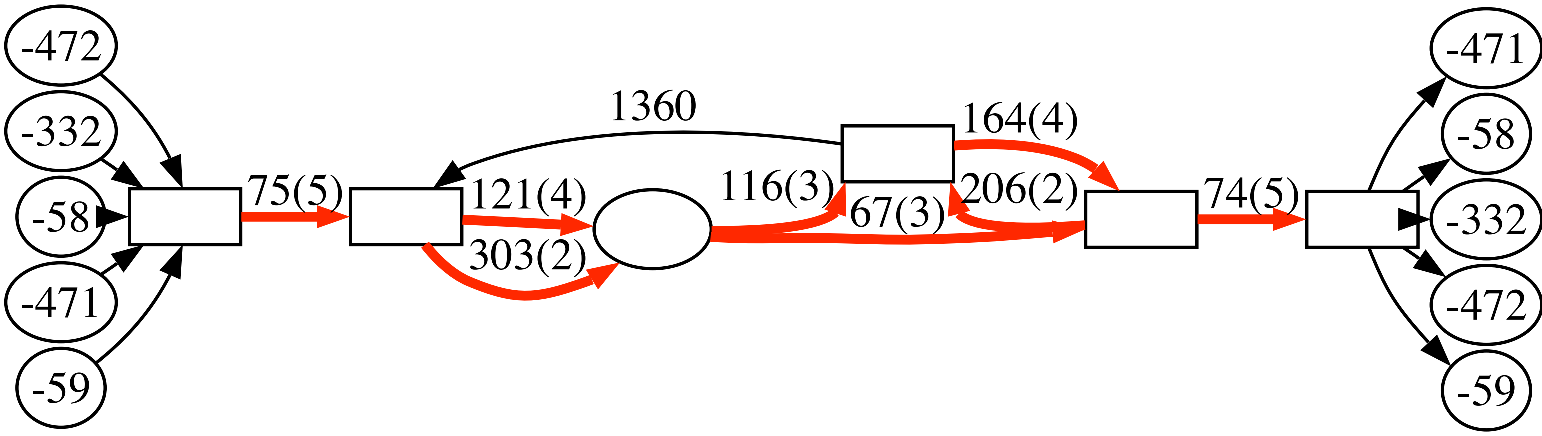

-472
-332
-58
-471
-59
75(5)
121(4)
303(2)
1360
116(3)
164(4)
67(3)
206(2)
74(5)
-471
-58
-332
-472
-59
